# Seasonal Variations of the Antioxidant Composition in Ground Bamboo *Sasa argenteastriatus* Leaves

**DOI:** 10.3390/ijms13022249

**Published:** 2012-02-20

**Authors:** Qinxue Ni, Guangzhi Xu, Zhiqiang Wang, Qianxin Gao, Shu Wang, Youzuo Zhang

**Affiliations:** 1 Agriculture and Food Science School, Zhejiang Agriculture and Forestry University, Lin’an 311300, China; E-Mails: niqinxue@hotmail.com (Q.N.); xuguangzhi3002@163.com (G.X.); wangzq0123@yahoo.com.cn (Z.W.); qianxingao@126.com (Q.G.); 2 School of Biosystems Engineering and Food Science, Zhejiang University, Hangzhou 310029, China; 3 Nurturing Station for the State Key Laboratory of Subtropical Silviculture, Zhejiang Agriculture and Forestry University, Lin’an 311300, China; E-Mail: 279934816@qq.com

**Keywords:** *Sasa argenteostriata*, leaves, seasonal variation, flavonoids, phenolics, triterpenoids, bamboo characteristic compounds, antioxidant activity

## Abstract

*Sasa argenteastriatus*, with abundant active compounds and high antioxidant activity in leaves, is a new leafy bamboo grove suitable for exploitation. To utilize it more effectively and scientifically, we investigate the seasonal variations of antioxidant composition in its leaves and antioxidant activity. The leaves of *Sasa argenteastriatus* were collected on the 5th day of each month in three same-sized sample plots from May 2009 to May 2011. The total flavonoids (TF): phenolics (TP) and triterpenoid (TT) of bamboo leaves were extracted and the contents analyzed by UV-spectrophotometer. Our data showed that all exhibited variations with the changing seasons, with the highest levels appearing in November to March. Antioxidant activity was measured using DPPH and FRAP methods. The highest antioxidant activity appeared in December with the lowest in May. Correlation analyses demonstrated that TP and TF exhibited high correlation with bamboo antioxidant activity. Eight bamboo characteristic compounds (orientin, isoorientin, vitexin, homovitexin and *p*-coumaric acid, chlorogenic acid, caffeic acid, ferulic acid) were determined by RP-HPLC synchronously. We found that chlorogenic acid, isoorientin and vitexin are the main compounds in *Sasa argenteastriatus* leaves and the content of isovitexin and chlorogenic acid showed a similar seasonal variation with the TF, TP and TT. Our results suggested that the optimum season for harvesting *Sasa argenteastriatus* leaves is between autumn and winter.

## 1. Introduction

Pharmacology studies have shown that many diseases of organisms such as aging, atherosclerosis, cancer, inflammation, *etc*. are related to oxidative damage caused by excess free radicals in body [1–3]. Free radicals are ions, atoms or molecules with unpaired electrons on an open shell configuration. In the human body, they can cause chain reactions with almost any inert substance under any inert conditions, resulting in cell death and tissue damage. Although almost all organisms possess antioxidant defense and repair systems that have evolved to protect them against oxidative damage, these systems are insufficient to prevent the damage entirely [4]. Therefore, exploration and development of free radical scavengers (antioxidants) from natural resources, and seeking methods of preventing and treating free radical-induced cardiovascular, cancer and other diseases by diet regulation has great significance. Various secondary metabolites, such as phenolics, flavonoids and triterpenoids, *etc*., widely distributed in many plants, have strong free radical scavenging capabilities [5–7].

Bamboo is a giant, woody grass mainly distributed in China, Japan and Southeast Asia, and is used as a building material and handicraft articles, as well as in the production of foodstuffs and traditional medicines. In particular, the leaves, as a kind of traditional Chinese medicine, are used for treating fever and as a detoxifier for more than 1000 years. Moreover, as a clinical traditional medicine, bamboo leaves are also used to cure or ameliorate stomach ache, diarrhea, vomiting, chest diaphragm inflammation, restlessness and excessive thirst [8]. A large number of studies have shown that the main biologically active component groups in bamboo leaves are firstly, *C*-glycoside flavonoids, represented by orientin, isoorientin, vitexin and homovitexin. Secondly, botanic phenolic acids, represented by *p*-coumaric acid, chlorogenic acid, caffeic acid and ferulic acid are also present [9,10]. These are all utilized as excellent for free radical scavenging, anti-oxidation, anti-aging, antibacterial and antiviral activity, anti-atherosclerosis, enhancing immunity, preventing degenerative diseases and other biological effectiveness. They also play an important role in resolving acrylic amide hazards in humans and ensuring food safety [11–14].

Until now, however, the utilization of bamboo leaves has focused on the family *Phyllostachys*. However, its small leaves and tall stem is problematic for leaf collection and limits utilization. Ground bamboos possessing developed underground whip and dense leaf structures have the advantages of rapid growth, lush foliage and easy collection. Our previous studies have compared the active components content and antioxidant activities of six different ground bamboo leaves. Results showed that the leaves of *Sasa argenteastriatus*, with an abundance of active compounds and high antioxidant activity, have the potential to be developed as a kind of leafy bamboo grove [15]. *Sasa argenteastriatus*, which belongs to the category *Sasa*, family *Gramineae*, is a kind of ground ornamental bamboo, mainly used for revetments and feed. Current studies have focused on investigation of its shooting and growth characteristics, and the determination of leaf nutrients [16–19]. The active ingredients in bamboo leaves and their physiological activities have rarely been reported. In order to improve the comprehensive utilization of bamboo leaves, this study reviews the total levels of phenolics (TP), flavonoids (TF) and triterpenoid (TT), plus eight characteristic compounds in bamboo leaves, in addition to antioxidant activity. We hypothesize that seasonal variations provoke changes in active secondary metabolites in bamboo leaves, further affecting its antioxidant activity. Therefore, we investigate antioxidant composition and activity as responses to seasonal changes. Simultaneously, we discuss the relationship between antioxidant activity and the amounts of TP, TF and TT, with the aim of providing a theoretical basis for exploiting and researching bamboo leaves more scientifically and efficiently.

## 2. Results and Discussion

### 2.1. Seasonal Variation of the Active Components Content in *Sasa argenteastriatu*s Leaves

**Figure 1:**
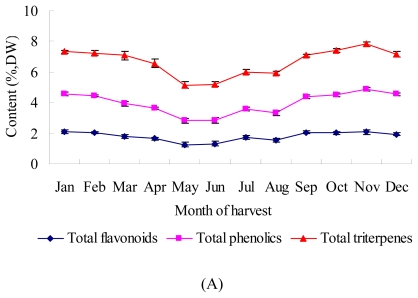

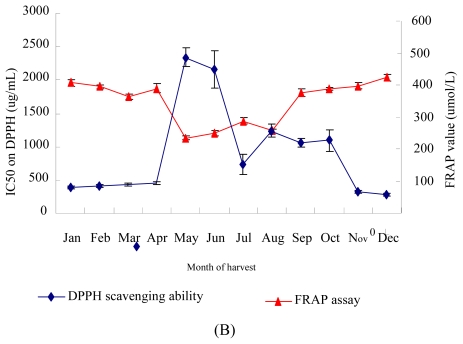
Seasonal variation of active components content and antioxidant activity of the leaves of *Sasa argenteastriatus.* (**A**) active components; (**B**) antioxidant activity (*n* = 6).

As a subtropical evergreen plant, bamboo has a two-year deciduous cycle. Active components in bamboo leaves have excellent biological effects: anti-oxidant, antibacterial, antiviral, immune regulation *etc.*, while the amounts of each component may vary with the seasons. The seasonal variation of various active components in bamboo leaves are shown in Figure 1A. TF content varied with seasonal change as a parabolic curve. The lowest TF content appeared in May, increasing gradually until arriving at a peak (2.10%) the following January, only to subsequently fall again. The trend of the seasonal variation in TP content was basically similar with TF, being generally higher in autumn-winter than in spring-summer. The content reached the highest point of the year (2.81%) in November, and then decreased gradually. The TT contents in *Sasa argenteastriatus* leaves were richer than those of TF and TP, but showing a similar trend, with higher content appeared in autumn-winter (peaking in November at 2.93%) and lower in spring-summer (with a minimum in May).

During spring and summer, the initial stages for the growth of shoots and leaves of bamboo, the content of secondary metabolites were at a low level. Afterwards, with growth and metabolism becoming slower, the flavonoids, phenolics and triterpenes were beginning to accumulate and maintain high levels throughout the autumn and winter. This is in line with the general law of transition and accumulation of secondary metabolites in plants [20,21].

### 2.2. Seasonal Variation in Antioxidant Activities of Bamboo Leaves

The scavenging ability of the 1,1-diphenylpicrylhydrazyl (DPPH) radical by different season bamboo leaf extracts was evaluated. The assays were carried out in methanol and the results expressed as IC_50_ (Figure 1B), which represents the antioxidant concentration necessary to scavenge the initial DPPH concentration by 50%. Lower IC_50_ indicated better DPPH radical scavenging ability. The DPPH radical scavenging ability of bamboo leaf extracts was significantly superior in autumn and winter than in spring and summer. It showed the strongest antioxidant ability from November to April (284.08~457.42 μg/mL), significantly stronger than that of other seasons, indicating an apparent relationship with the content of active components in bamboo leaves. This activity exhibits phenomena similar to that of bamboo from the family *Phyllostachys* [22].

FRAP assay measures the change in absorbance at 593 nm, due to the formation of a blue-colored Fe^2+^-tripyridyltriazine compound from the colorless, oxidized Fe^3+^ form by the action of electron donating antioxidants. The ability of bamboo leaf extracts harvested at differently seasons to reduce Fe^3+^ to Fe^2+^ ranged from 234.57 to 422.87 μmol/L at the concentration of 285.6 μg/mL (Figure 1B). The reduction in potency of bamboo leaf extracts harvested over four seasons decreased in the following order: winter > autumn > spring > summer.

### 2.3. Correlation Analysis

**Figure 2:**
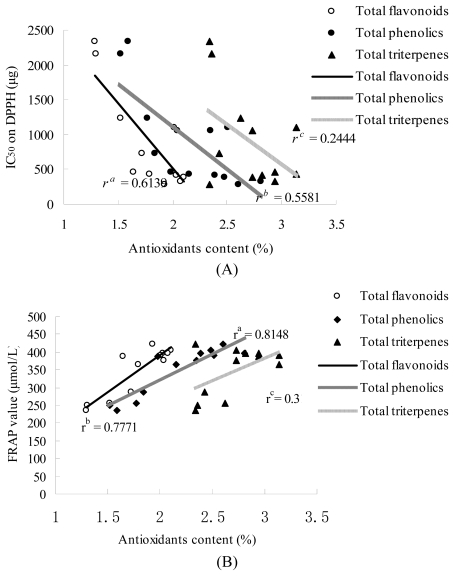
Correlation analysis between TF, TP, TT and IC_50_ on DPPH (**A**); FRAP value (**B**). (*n* = 6). ^a,b,c^ mean significant difference (*p* < 0.05).

Many studies show TF, TP and TT correlations with antioxidant properties [23–25]. Similar results were obtained in the present study (Figure 2A,B). Generally, the seasonal variation in the DPPH radical scavenging ability of bamboo leaves was consistent with changes in TF content. The correlation coefficient between TF, TP, TT and DPPH radical scavenging is TF > TP > TT, which indicated that TF in bamboo leaves play a leading role in the ability of DPPH radical scavenging. The correlation coefficient between TF, TP, TT and antioxidant ability obtained by FRAP assay is TP > TF > TT.

Flavonoids and phenolic, as botanic secondary metabolites, are major antioxidant components in plants [26,27]. Bamboo flavonoids in particular have been used in protecting human health with its certified antibacterial, blood pressure regulation, immune regulation and anti-aging activities. However, the distinct categories of active compounds in bamboo leaves and their amounts and biological activity were still vague and needed further research.

### 2.4. RP-HPLC Analyses of Eight Characteristics Compounds in Bamboo Leaves

Flavonoids and phenolics are the main antioxidant components in bamboo leaves [28,29]. For this reason, RP-HPLC parameters were optimized to analyze seasonal variation of the eight characteristic compounds in bamboo leaves, *i.e.*, orientin, isoorientin, vitexin, homovitexin and *p*-coumaric acid, chlorogenic acid, caffeic acid, ferulic acid (as shown in Table 1).

**Figure 3:**
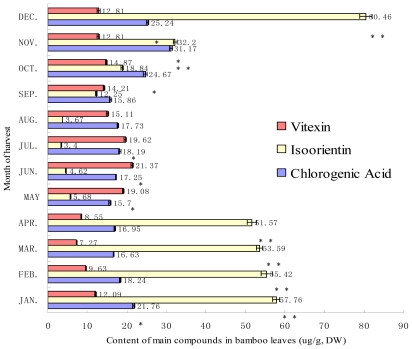
Seasonal variation of chlorogenic acid, isoorientin and vitexin in bamboo leaves (*n* = 6). * means *p <* 0.05; ** means *p <* 0.01.

The content of chlorogenic acid, isoorientin and vitexin were obviously more abundant than that of other five ingredients, and the trend was shown in Figure 3. The seasonal variation trend of four *C*-glycosides flavonoids was similar to TF with the exception of vitexin, which showed high content levels in autumn-winter. The content of isovitexin remained high from October to the following April (51.57~80.46 μg/g, DW), in contrast to May to September (Figure 3). The content of orientin and homovitexin showed slight changes with seasonal variations, 0.05~6.23 μg/g, DW and 0.58~1.63 μg/g, DW through the year respectively. Vitexin in bamboo leaves was abundant, and a seasonal variation trend not consistent with TF. Comparatively, the content of vitexin peaked in June (21.37 μg/g), reducing to a minimum in March (Figure 3).

Of the four characteristic phenolic acids found in bamboo, chlorogenic acid is the main phenolic acids in *Sasa argenteastriatus*, peaking in November at an annual maximum content (31.17 μg/g), then falling until May (Figure 3). Changes in levels of the other three phenolic acids with the seasons were slight.

**Figure 4:**
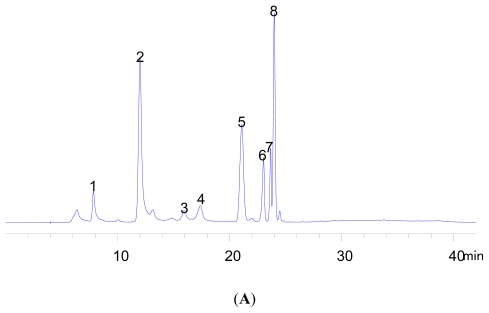

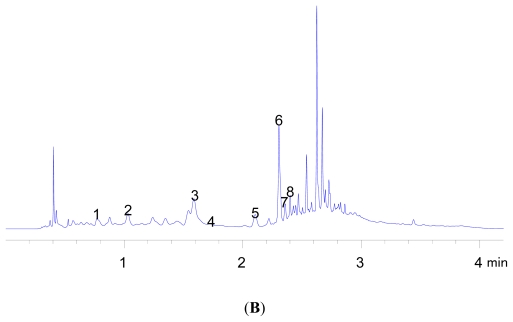
HPLC chromatograms of standard solution and extracts of bamboo leaves (**A**) Standard solution; (**B**) Sample of December. **1**: chlorogenic acid; **2**: caffeic acid; **3**: isoorientin; **4**: orientin; **5**: *p*-coumaric acid; **6**: vitexin; **7**: homovitexin; 8: ferulic acid.

Bamboo leaves are rich in various secondary metabolites. Supplements using bamboo leaf extract as the main functional component have played an important role in human healthcare. However, an understanding of the less-active compounds in bamboo leaves has been vague. As Figure 4 shows, many components in bamboo remain unknown and deserve further investigation.

## 3. Experimental Section

### 3.1. Plant Material Collection and Extraction

*Sasa argenteastriatus* leaves were collected at the same time (9 AM) on the 5th day of each month between May 2009 to May 2011 from the bamboo groves of Zhejiang Agricultural and Forestry University. Three identically-sized (1 × 1 m^2^) sample plots were randomly chosen in the groves. Leaves were picked randomly from different branches in each sample plot, carefully young and old leaves from the same sample plots. *Sasa argenteastriatus* was identified by bamboo taxonomy expert, Wei Fang of Zhejiang Agriculture and Forestry University. Bamboo leaves were washed, drained, and enzymes inactivated immediately (microwave treated at 640 W three times: 1 min each time), then dried in a vacuum drying oven (60 °C for 2 h), crushed, vacuum-packed, and stored in a ultra-low temperature refrigerator until all samples had been prepared. All samples from each month were investigated three times in order to decrease method-related measurement errors. The average results of six samples from each month over two successive years were used in the present study.

1 g of Bamboo leaf powder was mixed with 150 mL ethanol-aqueous solution (70%, v/v), refluxed 3 times (2 h each time), filtered and evaporated to constant volume of 100 mL. The moisture content of each sample was measured with an infrared moisture analyzer (OHAUS, Pine, NJ, USA) before extraction, and all the results were calculated based on dry materials.

### 3.2. Determination of TP, TF and TT Content

The TP concentration of the samples was determined using the Folin-Ciocalteu colorimetric method [30] with modifications. Briefly, a 0.2 mL sample of diluted bamboo leas extract was added to a tube and the volume made up to 10 mL using distilled water. After addition of 1 mL Folin-Ciocalteu reagent (Sigma-Aldrich Co., St. Louis, MO, USA) and 2.0 mL sodium carbonate solution (20%, w/v), the tube was placed in a boiling water bath for 1 min, and cooled at constant volume to 25 mL. The mixture was then blended and kept in the dark at room temperature for 30 min. The absorbance of the resulting blue color was measured at 745 nm using a T60 UV-vis spectrophotometer (Beijing Purkinje General Instrument Co., Ltd., Beijing, China) against a blank containing 0.2 mL of extraction solvent. The amount of TP was calculated as a *p*-hydroxybenzoic acid equivalent from the standard curve (*Y* = 15.564*X* + 0.0532, *r* = 0.9941), and expressed as the percentage of *p*-hydroxybenzoic in dry leaf weight (%, DW). All measurements were reproduced in triplicate.

The TF content of the samples was measured, based on the procedure described by Zhishen *et al*. and Mingyen *et al*. [31,32] with minor modifications, and using rutin (Sigma-Aldrich Co., St. Louis, MO, USA) as a standard. 0.5 mL of bamboo leaf extract was added to a tube and mixed with 0.3 mL sodium nitrite solution (5%, w/v). After standing for 5 min, the mixture was combined with 0.3 mL aluminum nitrate solution (10%, w/v). The mixture was blended and kept for 6 min before adding 2 mL sodium hydroxide solution (1.0 mol/L) and constant volume to 25 mL finally. Ten min later the absorbance of the solutions at 510 nm was measured against a blank containing 0.5 mL of extraction solvent. The amount of TF was calculated as a rutin equivalent from the standard curve (Y = 0.0062X – 0.0052, *r* = 0.9998), and expressed as the percentage of rutin in dry leaf weight (%, DW). All measurements were reproduced in triplicate.

The determination of TT content of the samples was performed as described by Xiang *et al*. and Yi *et al*. [33,34] with minor modification, and using ursolic acid as a standard. 0.5 mL of Bamboo leaf extract was transferred to a tube. After the solvent was heated to evaporation by nitrogen analyzer (Organomation Associates Inc., Berlin, MA, USA), 0.5 mL of vanillin-acetic acid solution (5%, w/v) and 0.8 mL perchloric acid were added, mixed and incubated at 65 °C for 15 min. The tubes were taken out and cooled in running water. Then 5 mL acetic acid was added. After being cooled to room temperature, with a blank solution as reference, the absorbance was scanned at 548 nm. The amount of TT was calculated as a ursolic acid equivalent from the standard curve (*Y* = 0.0048*X* + 0.0136, *r* = 0.9976), and expressed as the percentage of ursolic acid in dry leaf weight (%, DW). All measurements were reproduced in triplicate.

### 3.3. Determination of Antioxidant Activity

In the present study, antioxidant activity, in terms of diphenyl-2-picryl-hydrazyl (DPPH) assay, and ferric reducing antioxidant power (FRAP) assay were measured.

The DPPH free radical scavenging activity of the samples was measured using the method of Brand-William *et al*. and Sandeep *et al*. [35,36] with some modifications. The absorbance at 517 nm was measured 1 h after mixing 0.1 mL of bamboo leaf extracts (at a different concentration) with 3.9 mL DPPH solution (0.10 mmol/L), using a T60 UV-vis spectrophotometer (Beijing Purkinje General Instrument Co., Ltd., Beijing, China), using methanol as a blank. Triplicate tubes were prepared for each extract. The DPPH radical scavenging rate (%) was calculated in the following way: % radical scavenging rate = (A_blank_ – A_sample_)/A_blank_ × 100. Where A_blank_ is the absorbance of the blank reaction, and A_sample_ is the absorbance of the test compounds. The results were expressed as IC_50_ of DPPH radical scavenging, where IC_50_ means the amount of antioxidant necessary to decrease the initial concentration of DPPH radical (0.10 mmol/L) by 50%.

FRAP assay was performed following Benzie and Strain and Haichao *et al*. [37,38] with minor modifications. Briefly, the FRAP reagent contained 2.5 mL of 10 mmol/L tripyridyltriazine (TPTZ) solution in 40 mmol/L HCl, plus 2.5 mL of 20 mmol/L FeCl_3_ and 25 mL of 0.3 mol/L acetate buffer, pH 3.6, was freshly prepared. All bamboo leaf extracts were prepared at the same concentration (285.6 μg/mL). An aliquot of 0.3 mL of sample solution was mixed with 2.7 mL of FRAP reagent. The absorption of the reaction mixture was measured at 593 nm. 0.3 mL Methanol, and 2.7 mL TPTZ reagent mixture was used as a blank. Aqueous solutions of known Fe (II) concentration, in the range of 0–1000 μmol/L (FeSO_4_), were used for obtaining the calibration curve. Triplicate tubes were prepared for each extract. The reducing power of sample was expressed as an equivalent concentration of FeSO_4_. This FRAP parameter was defined as the concentration of antioxidant having a ferric reducing ability equivalent to that of 1 μmol FeSO_4_.

### 3.4. Eight Bamboo Characteristic Compounds Analysis by RP-HPLC

The amounts of eight bamboo characteristic compounds were analyzed using an HPLY system (consisting of a vacuum degasser, an auto-sampler, and a quaternary pump; Agilent Series 1200. Agilent Technologies Inc., Palo Alto, CA, USA) equipped with a reversed-phase C_18_ analytical column of 4.6 × 250 mm and 5 μm particle size (Inertsil^®^ ODS-SP). Column temperature was maintained at 40 °C. The injection volume was 10 μL. Mobile phase was acetonitrile (A); 1% acetic acid aqueous solution (B). The optimized chromatographic condition was: 0~15 min, A 15%, B 85%; 15~25 min, A 15%~40%, B 85%~60 %; 25~34 min, A 40%, B 60%; 34~40 min, A 40%~15%, B 60% ~ 85%; a 5 min post-run was used after each analysis. The flow rate used was 0.8 mL/min.

The standard 50% methanol-aqueous solution containing orientin 131 μg/mL, isoorientin 60 μg/mL, vitexin 55 μg/mL, homovitexin 50 μg/mL (Extrasynthese Chemical S.A.S., Lyon Nord, Genay Cedex, France), chlorogenic acid 55 μg/mL, caffeic acid 90 μg/mL, *p*-coumaric acid 75 μg/mL and ferulic acid 55 μg/mL (Sigma-Aldrich Co., St. Louis, MO, USA) was prepared. After diluting to make 5 solutions at different concentrations, the standard solution was filtered through a 0.45 μm filter and analyzed directly by HPLC. Curves of eight standard compounds using concentration as abscissa and peak area as the vertical axis were charted (Table 2).

All the bamboo leaf extracts were then passed again through a 0.45 μm filter and analyzed directly by HPLC. The amount of each compound in the bamboo leaves was calculated according to the standard curves.

Method evaluation was performed by the precision, repeatability, stability and recovery test. Precision was evaluated by analyzing the standard solution continuously seven times. The recovery test was carried out by adding known amounts of the stand sample to a preparation of bamboo leaf extract. The stability was evaluated by the analysis of bamboo leaf extract stored in a refrigerator at 2 h intervals over a period of 12 h. To confirm the repeatability of the method, seven preparations of bamboo leaf extract were analyzed repeatedly. The analysis of the bamboo leaf extracts was used for optimization of the analytical method (Table 2).

### 3.5. Statistical Analysis

Mean values and standard deviations were calculated from the data obtained from six samples each month. All the results are given as mean ± standard deviation (SD). Data were analyzed by one-way analysis of variance (ANOVA) [39]. Significant differences were assessed with as LSD test (*p* < 0.05). The statistical analysis was performed using the data collection program, SPSS [40].

## 4. Conclusions

The contents of active components *i.e.*, flavonoids, phenolics titerpenoids as well as antioxidant activity in *Sasa argenteastriatus* leaves change as a result of environment changes. Among the eight bamboo characteristic compounds, chlorogenic acid, iso-orientin and vitexin are the main active components. Meanwhile, a 70% ethanol-aqueous extract of bamboo leaves showed high antioxidant ability, as obtained by DPPH and FRAP assays. Correlation analysis showed TF in bamboo leaves play a leading role in the ability of DPPH radical scavenging, whereas FRAP relies mainly on TP in bamboo leaves. Thus *Sasa argenteastriatu*, with abundant active compounds and high antioxidant activity, has the potential to be exploited as a kind of leafy bamboo grove. Moreover, the present result may provide a theoretical basis for scientifically deciding optimal bamboo leaf-harvesting time and further exploitation of the leaf resources of the ground bamboo, *Sasa argenteostriata*. Further studies should focus on the health-related functionality and mechanism of the *Sasa argenteastriatus* leaves.

## Figures and Tables

**Table 1:** Seasonal variation of 5 less-important antioxidants in bamboo leaves (*n* = 6).

Month of Harvest	Caffeic Acid (μg/g, DW)	Orientin (μg/g, DW)	*p*-Coumaric Acid (μg/g, DW)	Homovitexin (μg/g, DW)	Ferulic Acid (μg/g, DW)
**January**	0.37 ± 0.003	1.78 * ± 0.012	0.98 * ± 0.005	1.04 * ± 0.018	0.17 ± 0.002
**February**	0.41 ± 0.008	0.98 * ± 0.007	0.73 ± 0.009	1.08 * ± 0.023	0.12 ± 0.001
**March**	0.39 ± 0.003	0.30 ± 0.002	0.41 ± 0.004	1.06 * ± 0.013	0.15 ± 0.001
**April**	0.37 ± 0.002	0.45 ± 0.004	0.50 ± 0.007	1.16 * ± 0.021	0.12 ± 0.001
**May**	0.72 ** ± 0.007	1.41 * ± 0.009	1.33 ** ± 0.007	0.58 ± 0.008	0.14 ± 0.001
**June**	0.64 ** ± 0.004	2.32 ** ± 0.016	1.47 ** ± 0.012	0.67 ± 0.006	0.17 ± 0.003
**July**	0.43 ± 0.003	3.04 ** ± 0.019	1.39 ** ± 0.019	0.79 ± 0.011	0.18 ± 0.003
**August**	0.47 * ± 0.004	2.76 ** ± 0.013	1.04 * ± 0.016	0.61 ± 0.005	0.18 ± 0.002
**September**	0.40 ± 0.008	4.55 ** ± 0.018	0.58 ± 0.008	1.19 * ± 0.020	0.39 * ± 0.009
**October**	0.41 ± 0.006	6.23 ** ± 0.025	0.48 ± 0.006	1.59 ** ± 0.025	0.44 * ± 0.006
**November**	0.39 ± 0.003	3.44 ** ± 0.037	0.55 ± 0.009	1.63 ** ± 0.017	0.34 * ± 0.005
**December**	0.38 ± 0.004	4.66 ** ± 0.052	0.88 * ± 0.013	1.55 ** ± 0.029	0.30 * ± 0.002

* *p <* 0.05;

** *p <* 0.01.

**Table 2:** Linear equations, correlation coefficients, precision, repeatability, stability and recovery analysis of eight compounds from bamboo leaves (*n* = 7).

Compounds	Retention Time (min)	Equations	*r*	Linear Range (μg/mL)	Precision, RSD%	Repeatability, RSD%	Stability, RSD%	Recovery%, RSD%
Chlorogenic acid	7.827	Y=73093X+0.957	0.9997	2.75~55.0	1.49	1.58	2.06	102.65, 1.94
Caffeic acid	11.985	Y=3E+06X-28.73	0.9965	4.5~90.0	0.93	1.03	1.29	101.97, 1.58
Isoorientin	15.927	Y=57642X-1.019	0.9993	3.0~60.0	1.32	0.89	1.07	99.21, 1.12
Orientin	17.366	Y=31371X+4.256	0.9979	6.55~131.0	1.17	1.27	1.54	103.11, 1.89
*p*-coumaric acid	21.069	Y=3E+06X+13.01	0.9979	3.75~75.0	1.86	1.51	1.87	98.26, 2.04
Vitexin	23.015	Y=1E+06X+9.252	0.9984	2.75~55.0	1.05	0.95	1.74	99.56, 1.75
Homovitexin	23.649	Y=1E+06X+3.812	0.9995	2.5~50.0	2.47	1.35	2.01	101.97, 1.47
Ferulic acid	23.971	Y=5E+06X+0.704	0.9999	2.75~55.0	0.93	1.16	1.96	97.75, 1.92
